# Outcomes and complications of primary rhegmatogenous retinal detachment repair with pars plana vitrectomy in young adults

**DOI:** 10.1186/s40942-023-00448-x

**Published:** 2023-02-22

**Authors:** Venkatkrish M. Kasetty, Jennifer Aye, Nish Patel, Nitika Tripathi, Thomas Hessburg, Nitin Kumar, Uday R. Desai, Abdualrahman E. Hamad

**Affiliations:** 1 Department of Ophthalmology, Henry Ford Health System, 2799 W. Grand Boulevard, Detroit, MI 48202 USA; 2 Murray Ocular Oncology and Retina, Miami, FL USA; 3 Department of Otolaryngology, Georgetown University, Washington, DC USA

**Keywords:** Rhegmatogenous retinal detachment, Pars plana vitrectomy, Proliferative vitreoretinopathy, Retinal reattachment

## Abstract

**Background:**

Scleral buckling has been the standard for rhegmatogenous retinal detachment repair in young patients given the typical lack of posterior vitreous detachment, phakic status, and lower risk of proliferative vitreoretinopathy. In older patients, pars plana vitrectomy alone is typically used for rhegmatogenous retinal detachment repair. We report the outcomes and complications of pars plana vitrectomy for rhegmatogenous retinal detachment in young eyes.

**Methods:**

Retrospective, single-center cohort study. Medical records of patients between 15 to 45 years of age undergoing primary pars plana vitrectomy for rhegmatogenous retinal detachment repair between 2010 and 2020 were carefully reviewed. All analyses were performed using the Kruskal–Wallis tests for numeric covariates between age groups.

**Results:**

Eyes were stratified by age: 15–24 (group 1, n = 10), 25–34 (group 2, n = 14), and 35–45 (group 3, n = 38). The average number of surgeries were 1.9, 1.4, and 1.1 in groups 1, 2, and 3, respectively (p = 0.004). Single surgery success rates were 50%, 64%, and 92% in groups 1, 2 and 3, respectively (p = 0.005). Final reattachment rates were 80%, 93%, 100% in groups 1, 2, and 3, respectively (p = 0.568). Proliferative vitreoretinopathy developed in 50%, 7%, and 8% of eyes in groups 1, 2, and 3, respectively (p < 0.001).

**Conclusion:**

While the final reattachment rates were excellent in all groups, the higher rates of proliferative vitreoretinopathy and lower single surgery success rate in younger patients may suggest that primary pars plana vitrectomy may not be the optimal repair method in these age groups.

## Background

Rhegmatogenous retinal detachments (RRD) are the most common type of retinal detachments and require urgent repair for good visual outcomes [1]. RRD incidence has a bimodal distribution; they typically occur in the elderly, with the highest incidence in the 60 to 70 age group, but there is another peak in the 20 to 30 age group in highly myopic patients [2]. The incidence widely ranges from 6.3 to 17.9 per 100,000 people and has increased by more than 50% in the last 2 decades [2, 3]. Risk factors for RRD include older age, male gender, high myopia, trauma, cataract surgery, lattice degeneration, family history, and prior RRD [1, 3]. In younger patients, RRD is typically caused by high myopia with atrophic holes, trauma, and vitreoretinal dystrophies such as Stickler’s Syndrome [4]. The detachments can be repaired using multiple techniques including scleral buckling (SB), pars plana vitrectomy (PPV), barrier laser, or pneumatic retinopexy. In some cases, multiple techniques are used [1]. The choice of surgical repair is surgeon and patient dependent, with PPV being the most common across all age groups [5, 6]. However, SB remains the preferred choice for RRD repair in the young adult population due to a typical phakic status and lack of posterior vitreous separation [7]. Recent studies have evaluated the outcomes of primary RRD repair in young adults with SB rates between 49 and 74% [8–11]. At our institution, some retina specialists perform PPV more often than SB in this age group. To our knowledge there have not been any studies evaluating outcomes of PPV in this young adult population stratified by age. We aim to analyze the anatomic and visual outcomes of PPV in the young adult population and to stratify this group by age to further analyze these outcomes.

## Methods

This is a retrospective cohort study of patients between the ages of 15 and 45 undergoing PPV for RRD repair between 2010 and 2020 at Henry Ford Health System in Michigan, USA. The institutional review board at Henry Ford Health System approved this study. This study adhered to the tenets of the Declaration of Helsinki. A Current Procedural Terminology (CPT) code of 67108 was used to identify patients. Exclusion criteria are listed in Table 1.

**Table 1:** Exclusion criteria

Exclusion criteria	Number excluded
Patients with follow-up less than 3 months or incomplete data in EHR	16
Penetrating trauma/open globe injury	9
Combined detachments (such as those secondary to proliferative diabetic retinopathy, retinal vascular disease, sickle cell retinopathy, other infectious or inflammatory causes)	8
RRD associated with giant retinal tear	4
Prior retinal surgery in the affected eye	1

Key: *EHR* electronic health record, *RRD* rhegmatogenous retinal detachment

Primary outcomes of this study include single surgery success (SSS) rate, the number of surgeries required for final reattachment, and reattachment rates. SSS was defined as only requiring one surgery for reattachment. Secondary outcomes included rates of posterior vitreous detachment (PVD) induction during surgery, proliferative vitreoretinopathy (PVR) and cataract formation, changes in visual acuity, and post-operative complications.

All analyses were performed using the Kruskal–Wallis tests for covariates between age groups. All analyses were performed using RStudio statistical software (RStudio, Boston, Massachusetts, USA).

## Results

A thorough search of the electronic health record between 2010 and 2020 revealed 102 eyes between the ages of 15 to 45 that had undergone PPV for RRD repair, of which 62 eyes qualified for the study. These 62 eyes were stratified by age: 15–24 (group 1, n = 10), 25–34 (group 2, n = 14), and 35–45 (group 3, n = 38). The average age at first PPV was 35.6 ± 8.7 with mean follow-up length of 990 days. Initial PPV was performed by one of four vitreoretinal surgeons.

### Baseline Characteristics

Baseline characteristics are presented in Table 2. PVD was present prior to the first PPV in 3/10 (30%), 5/14 (36%), and 31/38 (82%) of cases in groups 1, 2, and 3, respectively (p = 0.002). Myopia is defined as refraction between -6 and 0 diopters. High myopia is defined as a refraction ≤ -6 diopters. In group 1, 3/8 (37%) eyes were myopic and 5/8 (63%) were high myopes. In group 2, 3/9 (33%) eyes were myopic and 6/9 (67%) were high myopes. In group 3, 6/29 (21%) were myopic with 14/29 (48%) high myopes (p = 0.549 for myopia, 0.557 for high myopia).

**Table 2:** Baseline demographics of eyes undergoing primary vitrectomy for uncomplicated rhegmatogenous retinal detachments stratified by age (*denotes statistical significance)

	Average age	Gender	Macula status (off/on/partial)	PVD^a^ %	Myopia %	High myopia %
Group 1 (n = 10)	20.2	5 M, 5 F	7/3/0	3 (30)	3/8 (37)	5/8 (63)
Group 2 (n = 14)	30.4	5 M, 9 F	8/4/2	5 (36)	3/9 (33)	6/9 (67)
Group 3 (n = 38)	41.5	15 M, 23 F	23/12/3	31 (82)	6/29 (21)	14/29 (48)

Key: PVD (posterior vitreous detachment)

### Primary outcomes

**Fig. 1:**
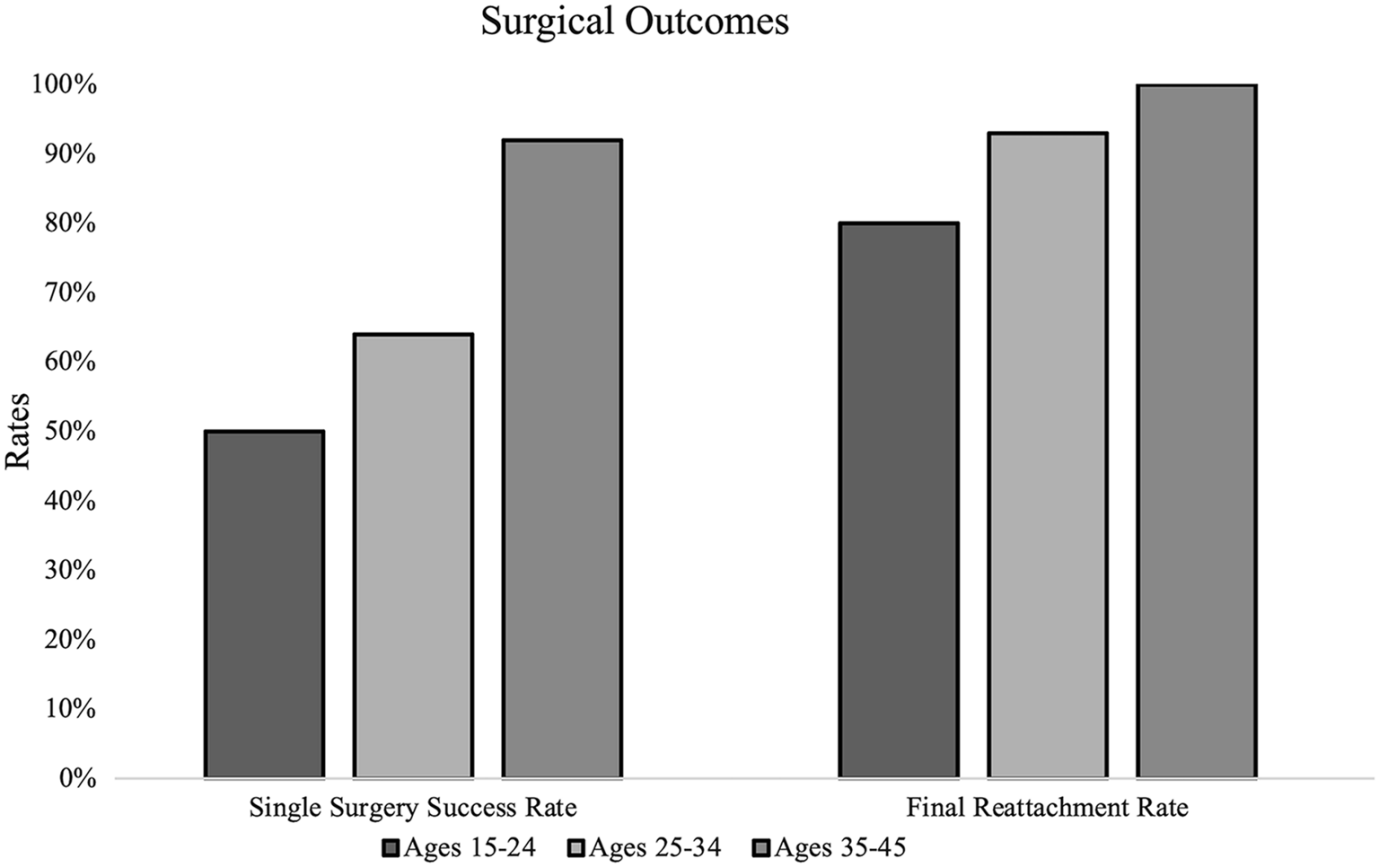
Surgical outcomes of eyes undergoing primary vitrectomy for uncomplicated rhegmatogenous retinal detachments stratified by age. Single surgery success rates were 50%, 64%, and 92% for groups 1, 2, and 3 respectively (p = 0.005). Final reattachment rates were 80%, 93%, 100% in groups 1, 2, and 3 respectively (p = 0.568)

Surgical outcomes are presented in Table 3. The average number of surgeries for reattachment in the groups 1, 2, and 3 were 1.9 (range: 1–4), 1.4 (range: 1–2), and 1.1 (range: 1–3) surgeries (p = 0.004), respectively. SSS rates for groups 1, 2, and 3 were 5/10 (50%), 9/14 (64%), and 35/38 (92%), respectively (p = 0.005, Fig. 1). Subsequent SB was performed in 3/5 (60%), 2/5 (40%), and 1/3 (33%) in groups 1, 2, and 3 (p = 0.737), respectively, for eyes with re-detachments. In group 1, 3 re-detachments were repaired with combined PPV/SB and 2 cases were repaired with PPV alone with silicone oil used in all cases. In group 2, 2 re-detachments were repaired with PPV/SB and 3 cases were repaired with PPV alone with silicone oil used in 3 of these cases. In group 3, 1 re-detachment was repaired with PPV/SB and 2 cases were repaired with PPV alone with silicone oil used in 2 of these cases. Total or near total RRDs were likely to re-detach after PPV with 6/12 (50%) requiring multiple surgeries and 6/13 (46%) of re-detachments being total or near-total RRDs. While less likely to re-detach than total or near total RRDs, 4/20 (25%) inferior RRDs re-detached with 4/13 (31%) re-detachments being inferior RRDs (Table 4). Final reattachment rates were 8/10 (80%), 13/14 (93%), and 38/38 (100%) in groups 1, 2, and 3 respectively (p = 0.568, Fig. 1). Two retinas in group 1 and one retina in group 2 remained detached. One eye in groups 1 and 2 were enucleated for blind, painful eyes.

**Table 3:** Surgical outcomes of eyes undergoing primary vitrectomy for uncomplicated rhegmatogenous retinal detachments stratified by age (^a^denotes statistical significance)

	SSS Rate^a^ %	Mean total number of surgeries^a^	Final reattachment rates %	SB placed at subsequent surgery %
Group 1	5 (50)	1.9 (range: 1–4)	8 (80)	3/5 (60)
Group 2	9 (64)	1.4 (range: 1–2)	13 (93)	2/5 (40)
Group 3	35 (92)	1.1 (range: 1–3)	38 (100)	1/3 (33)

Key: *VA* visual acuity, *SSS* single surgery success, *SB* scleral buckle

**Table 4:** Characteristics of detachments and surgical technique of eyes undergoing primary vitrectomy for uncomplicated rhegmatogenous retinal detachments

Case	Age	Retinal Break	Location of Detachment	PVD Presence	Needle Gauge	Initial Tamponade	Number of Surgeries	Subsequent Surgery	Subsequent Tamponade
Group 1									
1	15	Atrophic hole	Inferior	No	23	C_3_F_8_	3	PPV/SB	SO
2	18	Hole in lattice	Superior	No	23	C_3_F_8_	3	PPV	SO
3	19	Dialysis	Superotemporal	Yes	23	C_3_F_8_	1	–	–
4	20	Tear	Temporal	No	23	C_3_F_8_	1	–	–
5	21	Tear	Total	No	23	C_3_F_8_	2	PPV	SO
6	21	Dialysis	Temporal	No	23	C_3_F_8_	1	–	–
7	21	Tear	Superotemporal	Yes	23	C_3_F_8_	1	–	–
8	22	Hole in lattice	Inferotemporal	No	23	C_3_F_8_	1	–	–
9	22	Tear, atrophic hole	Inferior	No	23	C_3_F_8_	4	PPV/SB	SO
10	23	Operculated hole	Near Total	Yes	23	C_3_F_8_	2	PPV/SB	SO
Group 2									
1	25	Atrophic hole	Total	Yes	23	C_3_F_8_	2	PPV	SO
2	25	Hole in lattice	Inferior	No	23	C_3_F_8_	1	–	–
3	28	Tear	Near Total	No	23	C_3_F_8_	2	PPV/SB	SO
4	29	Tear	Inferotemporal	Yes	23	C_3_F_8_	1	–	–
5	30	Atrophic hole	Inferotemporal	No	23	C_3_F_8_	2	PPV/SB	C_3_F_8_
6	30	Hole in lattice	Inferior and Nasal	No	23	C_3_F_8_	1	–	–
7	31	Atrophic hole	Inferotemporal	No	23	C_3_F_8_	1	–	–
8	31	Atrophic hole	Superior	No	23	C_3_F_8_	1	–	–
9	32	Tear	Superior	Yes	23	C_3_F_8_	1	–	–
10	32	Hole in lattice	Inferior	No	23	C_3_F_8_	2	PPV	C_3_F_8_
11	32	Atrophic Hole	Superior	No	23	SF_6_	1	–	–
12	33	Hole in lattice	Inferior	No	23	C_3_F_8_	1	–	–
13	34	Atrophic hole	Superotemporal	Yes	23	C_3_F_8_	1	–	–
14	34	Tear	Near Total	Yes	23	C_3_F_8_	2	PPV	SO
Group 3									
1	36	Atrophic hole	Superotemporal	No	23	C_3_F_8_	1	–	–
2	36	Tear	Inferior	Yes	23	C_3_F_8_	1	–	–
3	36	Hole in lattice	Near Total	Yes	23	C_3_F_8_	1	–	–
4	36	Hole in lattice	Inferior	Yes	23	C_3_F_8_	1	–	–
5	37	Tear	Superior	Yes	23	C_3_F_8_	1	–	–
6	37	Tear	Superotemporal	Yes	23	C_3_F_8_	3	PPV	SO
7	37	Atrophic hole	Superotemporal	Yes	23	C_3_F_8_	1	–	–
8	38	Hole in lattice	Superior	Yes	23	C_3_F_8_	1	–	–
9	38	Tear	Near total	Yes	23	C_3_F_8_	1	–	–
10	38	Tear	Inferior	No	23	C_3_F_8_	1	–	–
11	40	Tear	Superonasal	Yes	23	C_3_F_8_	1	–	–
12	41	Tear	Inferior	Yes	23	C_3_F_8_	1	–	–
13	41	Tear	Near Total	Yes	23	C_3_F_8_	1	–	–
14	41	Hole in lattice	Inferior	No	23	C_3_F_8_	1	–	–
15	41	Tear, atrophic hole	Temporal	Yes	23	C_3_F_8_	2	PPV	C_3_F_8_
16	41	Hole in lattice	Inferotemporal	No	23	C_3_F_8_	1	–	–
17	42	Tear, hole in lattice	Near total	No	23	C_3_F_8_	1	–	–
18	42	Tear	Inferior	Yes	23	C_3_F_8_	1	–	–
19	42	Tear	Superotemporal	Yes	23	C_3_F_8_	1	–	–
20	42	No break found	Inferior	Yes	23	C_3_F_8_	1	–	–
21	43	Atrophic hole	Superonasal	Yes	25	–	1	–	–
22	43	Hole in lattice	Near total	Yes	23	C_3_F_8_	1	–	–
23	43	Hole in lattice	Inferior	Yes	23	C_3_F_8_	1	–	–
24	43	Tear	Near total	Yes	23	C_3_F_8_	2	PPV/SB	SO
25	43	Tear	Superior	Yes	23	SF_6_	1	–	–
26	44	Tear	Superior	Yes	23	C_3_F_8_	1	–	–
27	44	Tear	Superior	Yes	23	C_3_F_8_	1	–	–
28	44	Hole in lattice	Superotemporal	Yes	23	C_3_F_8_	1	–	–
29	44	Tear	Superior and Temporal	Yes	23	C_3_F_8_	1	–	–
30	44	Hole in lattice	Near Total	Yes	23	C_3_F_8_	1	–	–
31	44	Tear	Superotemporal	Yes	23	C_3_F_8_	1	–	–
32	45	Tear	Inferonasal	Yes	23	C_3_F_8_	1	–	–
33	45	Tear	Superior	Yes	23	C_3_F_8_	1	–	–
34	45	Tear	Superior	No	23	C_3_F_8_	1	–	–
35	45	No break found	Nasal	Yes	23	C_3_F_8_	1	–	–
36	45	Tear	Superior	Yes	23	C_3_F_8_	1	–	–
37	45	Tear	–	Yes	23	C_3_F_8_	1	–	–
38	45	Tear	Temporal	Yes	23	C_3_F_8_	1	–	–

Key: *C*_*3*_*F*_*8*_ Perfluoropropane, *SF*_6_ sulfur hexafluoride, *PPV* pars plana vitrectomy, *SB* scleral buckle, *SO* silicone oil

### Secondary outcomes

VA outcomes are presented in Table 5. Significant VA gains were seen in group 3 with an improvement in median VA from 20/100 to 20/32 compared to decreases of 20/63 to 20/80 and 20/40 to 20/50 seen in groups 1 and 2, respectively (p = 0.003). Only 4/10 (40%), 4/14 (29%), and 3/38 (8%) eyes had a final visual acuity of count fingers or worse in groups 1, 2, and 3 respectively. For the patients only requiring 1 surgery for reattachment, median VA improved in all three groups. Group 1 eyes improved from 20/40 to 20/25. Group 2 eyes improved from 20/40 to 20/20. Group 3 eyes improved from 20/80 to 20/25 (p = 0.177). For eyes with a macula-on RRD, there was no difference in VA improvements between groups (p = 0.128). For eyes with a macula-off RRD, significant VA gains were seen in group 2 eyes improving from a median VA of 20/100 to 20/70 and group 3 eyes improving from 20/320 to 20/32, while group 1 eyes decreasing from 20/80 to count fingers (p = 0.001).

**Table 5:** Visual acuity outcomes (logMAR [Snellen]) of eyes undergoing primary vitrectomy for uncomplicated rhegmatogenous retinal detachments stratified by age (^a^denotes statistical significance)

	All RRDs			Macula-On RRDs			Macula-Off RRDs		
	Median Initial VA	Median Final VA	Median Δ VA*	Median Initial VA	Median Final VA	Median Δ VA	Median Initial VA	Median Final VA	Median Δ VA^a^
Group 1	0.47 (20/63)	0.55 (20/80)	0.20	0.10 (20/25)	0.10 (20/25)	0	0.60 (20/80)	1.40 (CF)	0.40
Group 2	0.35 (20/40)	0.42 (20/50)	− 0.14	0.20(20/32)	0 (20/20)	− 0.09	0.70 (20/100)	0.54 (20/70)	− 0.09
Group 3	0.65 (20/100)	0.17 (20/32)	− 0.30	0.14 (20/25)	0 (20/20)	− 0.08	1.15 (20/320)	0.17 (20/32)	− 0.82

*VA* visual acuity, *RRD* rhegmatogenous retinal detachment, *CF* count fingers

A PVD was present prior to PPV in 3/10 (30%), 5/14 (36%), and 31/38 (82%) (p = 0.002). Of the patients without pre-operative PVD, a Weiss ring was induced in 5/7 (71%), 8/9 (88%), 5/7 (71%) in groups 1, 2 and 3, respectively. There was no significant difference in SSS or the number of surgeries required for reattachment when comparing eyes with pre-operative PVD to eyes without a pre-operative PVD in all 3 groups (Table 6). Two eyes in group 1, one eye in group 2, and one eye in group 3 did not have a PVD either present prior to PPV or induced during surgery. The PVD presence before and after PPV was unclear for the other eye.

**Table 6:** Outcomes of eyes undergoing primary vitrectomy for rhegmatogenous retinal detachment repair stratified by pre-operative PVD status

		SSS %	Number of surgeries
Group 1	PVD (n = 3)	2 (67)	1.3
	no PVD (n = 7)	3 (43)	2.1
Group 2	PVD (n = 5)	3 (60)	1.4
	no PVD (n = 9)	6 (67)	1.3
Group 3	PVD (n = 31)	29 (94)	1.1
	no PVD (n = 7)	6 (86)	1.3

*SSS* single surgery success, *PVD* posterior vitreous detachment

Post-operative complication outcomes are presented in Table 7. In group 1, cataracts developed in 5/10 (50%) eyes with extraction occurring in 3/5 (60%) eyes. Cataracts were first noted at a mean of 109 (range: 42 to 264) days after initial PPV with cataract extraction occurring at a mean of 267 (range: 49 to 645) days after initial PPV. In group 2, cataracts developed in 8/14 (57%) eyes with extraction occurring in 5/8 (63%) eyes. In group 2, cataracts were first noted at a mean of 103 (range: 21 to 304) days after initial PPV with cataract extraction occurring at a mean of 409 (range: 42 to 1253) days after initial PPV. In group 3, 6 eyes had already had cataract extraction and 3 eyes had documented cataracts prior to PPV. Cataracts developed in 20/29 (69%) eyes with extraction occurring in 13/20 (65%) eyes. Of the 3 eyes that had cataracts prior to PPV, cataracts progressed in 2 eyes with extraction occurring in one eye and lensectomy occurring at the time of initial PPV in the other eye. Cataracts were first documented at a mean of 369 (range: 2 to 2335) days after initial PPV with cataract extraction occurring at a mean of 973 (range: 49 to 3154) days after initial PPV. While group 3 had the highest rates of post-PPV cataract development, these rates were not statistically different between the groups (p = 0.516).

**Table 7:** Complications of eyes undergoing primary vitrectomy for uncomplicated rhegmatogenous retinal detachments stratified by age (^a^denotes statistical significance)

	Cataract development %	PVR development^a^	Glaucoma development^a^ %	Hypotony %^a^	Enucleation
Group 1	5/10 (50)	5 (50)	2 (20)	2 (20)	1
Group 2	8/14 (57)	1 (7)	2 (14)	3 (20)	1
Group 3	20/29 (69)	2 (8)	0 (0)	1 (3)	0

*PVR* proliferative vitreoretinopathy

Only 1 eye in group 3 had pre-operative PVR, which required 1 surgery for final reattachment. No other eyes had pre-operative PVR. Post-operative PVR rates were 5/10 (50%), 1/14 (7%) and 2/38 (8%) in groups 1, 2, and 3, respectively (p < 0.001). In group 1, 5/5 (100%) re-detachments were attributed to PVR. In group 2, 1/5 (20%) re-detachments were attributed to PVR. The other re-detachments were attributed to traction on the retinotomy site, missed break, incomplete initial PVD, and poor intraoperative patient cooperation. In group 3, 2/3 (67%) re-detachments were attributed to PVR and the other detachment was attributed to missed break at the vitreous base. One eye in groups 1, 2 and 3 each had a final VA of no light perception.

## Discussion

The optimal repair method for RRD has been discussed and debated extensively. In patients undergoing RRD repair, *Haugstad *et al*.* reported a PPV rate of 27% and a SB rate of 73% in patients between the ages of 0–40 [9]. *Brown *et al*.* did not perform primary PPV for RRD repair in patients between 18 and 30 years of age but instead preferred SB (71%) and combined PPV/SB (29%) with 74% and 64%, respectively [8]. Oftentimes, the repair method chosen is highly surgeon dependent, including when and where the surgeon trained and experiences after fellowship [12]. Recently, PPV has been preferred over SB in older patients due to better intraoperative wide-field visualization, a quicker operation and recovery, and decreased post-operative pain and inflammation [13–15]. In pediatric patients, it is generally accepted that SB results in optimal outcomes but it’s unknown at what age the success rates of PPV and SB becomes equivalent. A recent analysis of 2200 retinal detachments in children between the ages of 1 and 17 revealed that the best visual outcomes and SSS rates occur with SB compared to PPV and combined PPV/SB [16]. *Cai *et al*.* further demonstrated better SSS after SB or PPV/SB in young adult patients when compared to PPV, however in their study there was a preference towards SB (49% of repairs) and PPV/SB (30% of repairs) as primary repair modalities [11]. Our rates of PPV in the young adult population are consistent with PPV rates typically reported in older populations [9].

The young adult population shares similarities with both the pediatric population as well as the older population and provides unique challenges when undergoing PPV. The typical lack of complete PVD in this age group can make PPV challenging [17]. Inducing a PVD itself can be difficult in younger patients due to a strongly adherent vitreous, therefore the posterior hyaloid may not be able to be lifted to the vitreous base. Notably, the inability to induce a PVD makes it difficult or impossible to make a small posterior drainage retinotomy. Failing to induce a PVD and leaving residual hyaloid can result in re-detachment when the posterior hyaloid detaches over time or if the vitreous contracts [18]. As expected, a PVD was present prior to PPV in group 3 at higher rates (82%) compared to the groups 1 and 2 which had similar PVD rates (30% and 36%, respectively). In eyes that had a pre-operative PVD, SSS was achieved in 67%, 60%, and 94% of eyes in groups 1, 2, and 3 respectively. Of eyes that re-detached, a PVD was not present prior to PPV in 80%, 60%, and 0% in groups 1, 2, and 3 respectively. Two eyes in group 1, 1 eye in group 2, and 1 eye in group 3 did not have a PVD either present prior to PPV or induced during surgery. Two of these eyes (50%) required more than 1 surgery for reattachment. While not statistically significant, eyes with pre-operative PVD had a higher SSS (87%) compared to eyes without pre-operative PVDs (65%). Due to our small sample size, it is difficult to make conclusions on the effect of PVD presence on the visual outcomes and surgical success rates, but we suspect that the lack of a pre-operative PVD and failing to induce a PVD during PPV may increase the risk of re-detachment, especially in the younger cohorts.

In our study, PPV alone resulted in good anatomic success rates in these younger patients. While our overall SSS rate was 79% and higher than previously reported in the literature, it is skewed towards our oldest age group [4, 19]. SSS rates were significantly higher in group 3 (92%) compared to groups 1 (50%) and 2 (64%), indicating that PPV is less likely to be a “one and done” repair method in younger patients. Consequently, group 1 required almost twice as many surgeries for final reattachment compared to group 3 (1.9 vs. 1.1, respectfully. If a subsequent surgery was required, a combined PPV/SB was performed in 60%, 40%, and 33% of eyes in groups 1, 2, and 3 respectively, indicating a preference for adding a SB in younger patients after PPV failure. Additionally, inferior and total or near total RRD were most likely to re-detach compared to other locations of RRDs after primary PPV, which may be an additional risk factor due to potential non-compliance issues related to post-operative patient positioning. Final reattachment rates were good in all groups favoring group 3.

Visual outcomes favored our oldest patients as well. VA gains were only seen in patients aged 35–45 with decreases seen in groups 1 and 2. In eyes with SSS, however, VA improvements were seen in all groups. In macula-on RRDs, final VA was excellent with improvements seen in groups 2 and 3. As expected, in macula-off RRDs, final VA outcomes were worse than macula-on RRDs, however, VA improvements were seen in groups 2 and 3, with a VA decrease seen in group 1. The average number of surgeries for reattachment for macula-on and macula-off RRDs was 1.1 and 1.4, respectively. The lack of visual improvement in younger patients is likely due to the increased rates of PVR and increased number of reoperations required for reattachment with a decreased visual potential following each reoperation.

PVR formation is a potentially vision-threatening complication of RRD and can lead to retinal re-detachment. PVR occurred infrequently in our study cohort with an overall rate of 13%, but at higher rates in our youngest group. Our overall PVR rates are consistent with rates reported in the literature and better than those reported by *Brown *et al. with a combined rate of 35% in SB and PPV/SB eyes [8, 20]. While our study did not compare PVR rates between RRD repair methods, the higher PVR rates in our younger patients may not be directly attributed to PPV alone as these younger eyes may be more prone to PVR and may have developed PVR with any repair modality [21].

Another known complication of PPV is cataract development as these younger patients are typically phakic [22, 23]. In our study, cataract development rates were similar amongst the three groups with an average rate of 62%. Cataracts developed and were removed sooner in the younger groups compared in group 3. This could indicate that cataracts were more aggressively removed, became visually significant sooner, or the cataract may have needed to be removed in order for a better view for subsequent retinal surgery. Previous studies have described that most cataracts developed within 1 year of PPV in patients below 30, which is consistent with our data [22].

The main limitations of this study are its small sample size and retrospective nature. As in any retrospective study, incomplete data due to inconsistent follow-up as well as a lack of standardized data documentation can lead to confounding variables. Additionally, our study does not compare primary PPV to SB or PPV/SB.

## Conclusions

While the rates of cataract formation were similar between the groups and final reattachment rates were good, the higher rates of PVR and lower SSS rates in the younger patients may suggest that primary PPV in these patients may not be the best surgical modality in patients below 35 years of age. However, we cannot make this statement definitively since we do not compare groups of similarly aged patients undergoing SB alone and therefore, we cannot say whether these worse outcomes in the younger groups are related to the variables related to their age or to the method of repairing the detachment. In these younger patients, other repair methods should be explored. Further prospective studies or big-data retrospective studies on PPV in the young adult population should be performed to make a more definitive conclusion on its utility in this population.

## Meeting presentation

This work was presented on October 9th, 2021 at the American Society for Retinal Specialists 2021 Annual Meeting.

## Data Availability

The datasets used and/or analyzed during the current study are available from the corresponding author on reasonable request.

## Abbreviations

RRD: Rhegmatogenous retinal detachment
SB: Scleral buckling
PPV: Pars plana vitrectomy
SSS: Single surgery success rate
PVD: Posterior vitreous detachment
PVR: Proliferative vitreoretinopathy

## References

[1] Vail D, Pan C, Pershing S, Mruthyunjaya P (2020). Association of rhegmatogenous retinal detachment and outcomes with the day of the week that patients undergo a repair or receive a diagnosis. JAMA Ophthalmology.

[2] Mitry D, Charteris DG, Fleck BW, Campbell H, Singh J (2010). The epidemiology of rhegmatogenous retinal detachment: geographical variation and clinical associations. Br J Ophthalmol.

[3] Nielsen BR, Alberti M, Bjerrum SS, la Cour M (2020). The incidence of rhegmatogenous retinal detachment is increasing. Acta Ophthalmol.

[4] Lakehal-Ayat Y, Angioi K, Berrod JP, Conart JB (2020). Rhegmatogenous retinal detachment in young adults: Clinical characteristics and surgical outcomes. J Fr Ophtalmol.

[5] Reeves M-G, Pershing S, Afshar AR (2018). Choice of primary rhegmatogenous retinal detachment repair method in us commercially insured and medicare advantage patients, 2003–2016. Am J Ophthalmol.

[6] Vail D, Pershing S, Reeves MG, Afshar AR (2020). The relative impact of patient, physician, and geographic factors on variation in primary rhegmatogenous retinal detachment management. Ophthalmology.

[7] Schaal S, Sherman MP, Barr CC, Kaplan HJ (2011). Primary retinal detachment repair: comparison of 1-year outcomes of four surgical techniques. Retina.

[8] Brown K, Yannuzzi NA, Callaway NF, Patel NA, Relhan N, Albini TA (2019). Surgical outcomes of rhegmatogenous retinal detachment in young adults ages 18–30 years. Clin Ophthalmol.

[9] Haugstad M, Moosmayer S, Bragadόttir R (2017). Primary rhegmatogenous retinal detachment - surgical methods and anatomical outcome. Acta Ophthalmol.

[10] Safadi K, Chowers I, Khateb S (2021). Outcomes of primary rhegmatogenous retinal detachment repair among young adult patients. Acta Ophthalmol.

[11] Cai L, Ammar MJ, Ryan E, Wang J, Ryan C, Wu CM (2020). Anatomic and visual outcomes of primary retinal detachment in younger adults a report from the multicenter primary retinal detachment outcomes (PRO) study. Invest Ophthalmol Visual Sci.

[12] D’Amico D, Tornabe P, Edwin R. Controversies in care: Rhegmatogenous retinal detachment repair retinal physician: Pentavision; 2011. https://www.retinalphysician.com/issues/2011/jan-feb/controversies-in-care.

[13] Khanduja S, Kakkar A, Majumdar S, Vohra R, Garg S (2013). small gauge vitrectomy: recent update. Oman J Ophthalmol.

[14] Mohamed YH, Ono K, Kinoshita H, Uematsu M, Tsuiki E, Fujikawa A (2016). Success rates of vitrectomy in treatment of rhegmatogenous retinal detachment. J Ophthalmol.

[15] Thompson JT (2011). Advantages and limitations of small gauge vitrectomy. Surv Ophthalmol.

[16] Yonekawa YS, Wakabayashi M, Sharma T, Boucher C, Klufas N, Spirin M (2022). State of pediatric retinal detachment surgery in the United States: an aggregated electronic health record analysis of 2200 Children.

[17] Syed Z, Stewart MW (2016). Age-dependent vitreous separation from the macula in a clinic population. Clin ophthalmol.

[18] Wa C, Yee K, Huang L, Sadun A, Sebag J (2013). Long-term safety of vitrectomy for patients with floaters. Invest Ophthalmol Visual Sci.

[19] Marques JH, Castro C, Malheiro L, Alves Correia N, Pessoa B, Melo Beirão J (2021). Dealing with rhegmatogenous retinal detachment in patients under 40 years old: a tertiary center results. Int Ophthalmol.

[20] Idrees S, Sridhar J, Kuriyan AE (2019). Proliferative vitreoretinopathy: a review. Int Ophthalmol Clin.

[21] Ciprian D (2015). The pathogeny of proliferative vitreoretinopathy. Rom J Ophthalmol.

[22] Blodi BA, Paluska SA (1997). Cataract after vitrectomy in young patients. Ophthalmology.

[23] Feng H, Adelman RA (2014). Cataract formation following vitreoretinal procedures. Clin Ophthalmol.

